# Analytical Model for Concentration (Pressure) Impedance of a Low-Pt PEM Fuel Cell Oxygen Electrode

**DOI:** 10.3390/membranes12040356

**Published:** 2022-03-24

**Authors:** Andrei Kulikovsky

**Affiliations:** Theory and Computation of Energy Materials (IEK-13), Institute of Energy and Climate Research, Forschungszentrum Jülich GmbH, D-52425 Jülich, Germany; a.kulikovsky@fz-juelich.de

**Keywords:** PEM fuel cell, low-Pt loaded cathode, concentration impedance, pressure impedance, modeling

## Abstract

In this study, a model for concentration/pressure impedance ζ of the cathode catalyst layer of a low-Pt PEM fuel cell is developed. The model is based on transient oxygen mass transport equations through the cathode catalyst layer modeled as a single pore with a thin Nafion film covering the pore surface. This structure is used to simulate oxygen transport through the catalyst layer depth and through the ionomer film covering Pt/C agglomerates in low-Pt cells. Analytical solution for zeta-impedance at high cell current is derived; this solution can be used for fast fitting of experimental zeta-spectra. Optimal conditions for measuring the zeta-spectra of a low-Pt cell are discussed. Zeta impedance is not affected by faradaic processes in the cell, which makes this technique a useful alternative to standard EIS.

## 1. Introduction

Lowering of Pt loading is of large importance for the success of PEM fuel cells on the mass market. However, the first attempts to lower Pt loading on the cathode side have already revealed unexpected “overlinear” transport loss of the cell potential [1,2]. This effect has been attributed to oxygen transport through a thin ionomer film covering Pt/C agglomerates in the cathode catalyst layer (CCL). Over the past decade, a lot of research has been done to measure the film transport resistivity $R_{N}$ in PEMFCs [2,3,4,5,6,7,8,9]. Most of the works employed a limiting current method for measuring $R_{N}$, moreover, in [9] hydrogen instead of oxygen was used in the limiting current experiments to avoid undesired effects due to oxygen reduction reaction (ORR) in the electrode.

An alternative method, requiring standard cell, equipment, and procedures, is electrochemical impedance spectroscopy (EIS). A numerical model for low-Pt PEMFC impedance has been developed and fitted to the experimental impedance spectra [10]. Fitting returned the Nafion film thickness and oxygen diffusivity. Nonetheless, complexity of the impedance model [10] stimulates searching for simpler ways for measuring $R_{N}$. Generally, every transport process in a fuel cell is expected to form a separate peak in the distribution of relaxation times (DRT) spectrum [11]. The area under the DRT peak gives the contribution of the respective process to the total cell polarization resistivity. Thus, calculation of $R_{N}$ from experimental impedance spectra would be much simpler if the DRT peak due to oxygen transport in Nafion film were well separated from other peaks. However, a recent model [12] shows that at low currents, the characteristic frequency of the film-transport peak is only 1.73 times less than the frequency of faradaic peak and the two peaks tend to merge. An impedance spectroscopy method insensitive to faradaic processes in the cell would be of great help.

The idea to perturb cell potential by harmonic variation of the oxygen concentration or pressure (EPIS) has been suggested in [13] and developed further in [14,15,16,17,18]. A review of pressure impedance works can be found in [16]. The great advantage of this technique is that the zeta-spectrum of the cell is independent of the faradaic processes. So far, most of the reported EPIS models have been numerical. An analytical model for the PEMFC zeta-impedance taking into account oxygen transport in the gas diffusion layer (GDL) and CCL has been derived in [19]; however, this mean-field model ignores oxygen transport in ionomer film covering Pt/C agglomerates.

In this work, we report a model for the concentration impedance ζ of a low-Pt cell, with explicit account of oxygen transport through the ionomer film. We derive a formula for the ζ-impedance of a low-Pt cell suitable for fast fitting of experimental zeta-spectra. We show that measurements of the oxygen diffusion coefficient in the Nafion film using zeta-spectroscopy should be done at low oxygen concentration providing high rate of oxygen consumption in the CCL and at the cell current density close to the limiting current density due to Nafion film.

## 2. Model

### 2.1. Basic Transient Equations

It is assumed that the following conditions hold

- Oxygen transport loss in the gas-diffusion layer is small;
- Proton transport in the CCL is fast;
- Oxygen consumption in the CCL is large.

The limiting current density due to oxygen transport in the GDL is typically much larger, than the limiting current due to Nafion film [20] and to a first approximation oxygen transport loss in the GDL can be neglected. The second and third assumptions are discussed in Section 3.

**Figure 1:**
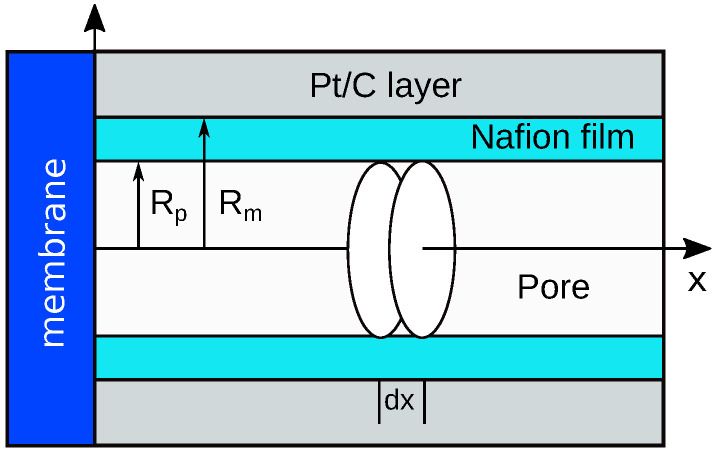
Schematic of a single-pore model for impedance of the low-Pt cathode catalyst layer.

The low–Pt CCL is modeled by a single cylindrical pore penetrating through the whole CCL depth. The pore volume is separated from the coaxial Pt/C tube by a thin Nafion film (Figure 1). Oxygen is transported along the pore and in the radial direction through the ionomer to Pt surface, where the ORR occurs. The static version of this model has been reported in [20]; here we briefly repeat the basic equations necessary for understanding the impedance model.

Oxygen transport along the pore is described by

(1) $$\frac{\partial c}{\partial t}-D_{p}\frac{\partial^{2}c}{\partial x^{2}}=\frac{2N_{N,p}}{R_{p}},\;{\left.\frac{\partial c}{\partial x}\right|}_{x=0}=0,\;c\left(l_{t}\right)=c_{1}$$

where $R_{p}$ is the pore radius, $D_{p}$ is the oxygen diffusion coefficient in the pore, *c* is the oxygen concentration in the pore, $c_{1}$ is the oxygen concentration at the pore/GDL interface and

(2) $$N_{N,p}={\left.D_{N}\frac{\partial c_{N}}{\partial r}\right|}_{r=R_{p}}$$

is the radial oxygen flux in the Nafion film at the pore/film interface.

Radial oxygen transport through the film is described by the diffusion equation

(3) $$\begin{array}{c} \frac{\partial c_{N}}{\partial t}-\frac{D_{N}}{r}\frac{\partial }{\partial r}\left(r\frac{\partial c_{N}}{\partial r}\right)=0,\;c_{N}\left(R_{p}\right)=K_{H}c\left(x\right),\\ {\left.D_{N}\frac{\partial c_{N}}{\partial r}\right|}_{r=R_{m}}=-\frac{R_{p}i_{*}}{2(4F)}\left(\frac{c_{N,m}}{c_{ref}}\right)\exp\left(\frac{\eta }{b}\right) \end{array}$$

where $c_{N}$ is the oxygen concentration in the Nafion film, $c_{N,m}\equiv c_{N}\left(R_{m}\right)$, $D_{N}$ is the oxygen diffusion coefficient in the film, $i_{*}$ is the ORR exchange current density, η is the positive by convention ORR overpotential, and *b* is the ORR Tafel slope. The left boundary condition for Equation (3) is Henry’s law for oxygen dissolution in Nafion. The right boundary condition to Equation (3) describes consumption of dissolved oxygen in the ORR; the factor $R_{p}/2$ provides correct transition to the standard oxygen mass conservation equation in the CCL for the limiting case of zero Nafion film thickness.

To simplify the calculations we introduce dimensionless variables

(4) $$\begin{array}{c} \tilde{x}=\frac{x}{l_{t}},\;\tilde{R}=\frac{r}{l_{t}},\;\tilde{t}=\frac{ti_{*}}{4Fc_{ref}},\;\tilde{\eta }=\frac{\eta }{b},\;\tilde{j}=\frac{j}{i_{*}l_{t}},\\ \;\tilde{D}=\frac{4FDc_{ref}}{i_{*}l_{t}^{2}},\;\tilde{\omega }=\frac{\omega 4Fc_{ref}}{i_{*}},\;\tilde{\zeta }=\frac{\zeta c_{ref}}{b} \end{array}$$

where $l_{t}$ is the pore length (CCL thickness), ω is the angular frequency of AC signal, and ζ is the concentration impedance (see below).

With Equation (4), Equations (1) and (3) transform to

(5) $$\frac{\partial \tilde{c}}{\partial \tilde{t}}-{\tilde{D}}_{p}\frac{\partial^{2}\tilde{c}}{\partial {\tilde{x}}^{2}}=\frac{2{\tilde{N}}_{N,p}}{{\tilde{R}}_{p}}$$

(6) $$\begin{array}{c} \frac{\partial {\tilde{c}}_{N}}{\partial \tilde{t}}-\frac{{\tilde{D}}_{N}}{\tilde{r}}\frac{\partial }{\partial \tilde{r}}\left(\tilde{r}\frac{\partial {\tilde{c}}_{N}}{\partial \tilde{r}}\right)=0,\;{\tilde{c}}_{N}\left({\tilde{R}}_{p}\right)=K_{H}\tilde{c}\left(\tilde{x}\right),\\ {\left.{\tilde{D}}_{N}\frac{\partial {\tilde{c}}_{N}}{\partial \tilde{r}}\right|}_{\tilde{r}={\tilde{R}}_{m}}=-\frac{{\tilde{R}}_{p}}{2}{\tilde{c}}_{N,m}\exp\left(\tilde{\eta }\right) \end{array}$$

Systems (5) and (6) form the basis for the concentration impedance model.

### 2.2. Equations for Perturbation Amplitudes

Now we apply small-amplitude perturbations of the form

(7) $$\begin{array}{cc} \; & \tilde{c}\left(\tilde{x},\tilde{t}\right)={\tilde{c}}^{0}\left(\tilde{x}\right)+{\tilde{c}}^{1}\left(\tilde{x},\tilde{\omega }\right)\exp\left(i\tilde{\omega }\tilde{t}\right)\\ \; & {\tilde{c}}_{N}\left(\tilde{x},\tilde{t}\right)={\tilde{c}}_{N}^{0}\left(\tilde{x}\right)+{\tilde{c}}_{N}^{1}\left(\tilde{x},\tilde{\omega }\right)\exp\left(i\tilde{\omega }\tilde{t}\right)\\ \; & \tilde{\eta }\left(\tilde{t}\right)={\tilde{\eta }}^{0}+{\tilde{\eta }}^{1}\left(\tilde{\omega }\right)\exp\left(i\tilde{\omega }\tilde{t}\right). \end{array}$$

where the superscripts 0 and 1 mark the static functions and the perturbation amplitudes, respectively. Note that fast proton transport means that the static and perturbed ORR overpotentials are independent of $\tilde{x}$.

Substituting Equation (7) into Equations (5) and (6) and performing standard procedure of linearization, we come to the system of linear equations for ${\tilde{c}}^{1}$ and ${\tilde{c}}_{N}^{1}$:

(8) $${\tilde{D}}_{p}\frac{\partial^{2}{\tilde{c}}^{1}}{\partial {\tilde{x}}^{2}}=-\frac{2{\tilde{N}}_{N,p}^{1}}{{\tilde{R}}_{p}}+i\tilde{\omega }{\tilde{c}}^{1},\;{\left.\frac{\partial {\tilde{c}}^{1}}{\partial \tilde{x}}\right|}_{\tilde{x}=0}=0,\;{\tilde{c}}^{1}\left(1\right)={\tilde{c}}_{1}^{1}$$

(9) $$\begin{array}{c} \frac{{\tilde{D}}_{N}}{\tilde{r}}\frac{\partial }{\partial \tilde{r}}\left(\tilde{r}\frac{\partial {\tilde{c}}_{N}^{1}}{\partial \tilde{r}}\right)=i\tilde{\omega }{\tilde{c}}_{N}^{1},\;{\tilde{c}}_{N}^{1}\left({\tilde{R}}_{p}\right)=K_{H}{\tilde{c}}^{1}\left(\tilde{x}\right),\\ {\left.{\tilde{D}}_{N}\frac{\partial {\tilde{c}}_{N}^{1}}{\partial \tilde{r}}\right|}_{\tilde{r}={\tilde{R}}_{m}}=-\frac{{\tilde{R}}_{p}}{2}e^{{\tilde{\eta }}^{0}}\left({\tilde{c}}_{N}^{1}\left({\tilde{R}}_{m}\right)+{\tilde{c}}_{N,m}^{0}{\tilde{\eta }}^{1}\right), \end{array}$$

where ${\tilde{c}}_{N,m}^{0}={\tilde{c}}_{N}^{0}\left({\tilde{R}}_{m}\right)$, ${\tilde{c}}_{1}^{1}$ is the oxygen perturbation at the pore/GDL interface, and

(10) $${\tilde{N}}_{N,p}^{1}={\left.{\tilde{D}}_{N}\frac{\partial {\tilde{c}}_{N}^{1}}{\partial \tilde{r}}\right|}_{\tilde{r}={\tilde{R}}_{p}}$$

is the perturbed oxygen flux in the Nafion film at the pore interface. Note that due to assumption of fast oxygen transport in the GDL we have

(11) $${\tilde{c}}_{1}^{1}={\tilde{c}}_{h}^{1},$$

i.e., ${\tilde{c}}_{1}^{1}$ is equal to the applied oxygen concentration perturbation in the channel ${\tilde{c}}_{h}^{1}$.

The system of Equations (8) and (9) with the coefficient functions given by Equations (13) and (14) in the next section determine the CCL concentration impedance $\tilde{\zeta }$

(12) $$\tilde{\zeta }=\frac{{\tilde{\eta }}^{1}}{{\tilde{c}}_{h}^{1}}.$$

Here, ${\tilde{c}}_{h}^{1}$ is the oxygen concentration perturbation applied in the gas channel, and ${\tilde{\eta }}^{1}$ is the measured perturbation of the cell potential.

### 2.3. Static Equations and Solutions

Static equations for ${\tilde{c}}^{0}$ and ${\tilde{c}}_{N}^{0}$ are obtained from Equations (5) and (6) simply by chalking out the time derivatives:

(13) $$-{\tilde{D}}_{p}\frac{\partial^{2}{\tilde{c}}^{0}}{\partial {\tilde{x}}^{2}}=\frac{2}{{\tilde{R}}_{p}}{\left.{\tilde{D}}_{N}\frac{\partial {\tilde{c}}_{N}^{0}}{\partial \tilde{r}}\right|}_{\tilde{r}={\tilde{R}}_{p}},\;{\left.\frac{\partial {\tilde{c}}^{0}}{\partial \tilde{x}}\right|}_{\tilde{x}=0}=0,\;{\tilde{c}}^{0}\left(1\right)={\tilde{c}}_{1}$$

(14) $$\frac{{\tilde{D}}_{N}}{\tilde{r}}\frac{\partial }{\partial \tilde{r}}\left(\tilde{r}\frac{\partial {\tilde{c}}_{N}^{0}}{\partial \tilde{r}}\right)=0,\;\;{\tilde{c}}_{N}^{0}\left({\tilde{R}}_{p}\right)=K_{H}{\tilde{c}}^{0}\left(\tilde{x}\right),\;{\left.{\tilde{D}}_{N}\frac{\partial {\tilde{c}}_{N}^{0}}{\partial \tilde{r}}\right|}_{\tilde{r}={\tilde{R}}_{m}}=-\frac{{\tilde{R}}_{p}}{2}{\tilde{c}}_{N,m}^{0}\exp{\tilde{\eta }}^{0}$$

The solution to Equation (14) is

(15) $${\tilde{c}}_{N}^{0}\left(\tilde{r}\right)=\left(\frac{{\tilde{R}}_{p}{\tilde{R}}_{m}\ln\left({\tilde{R}}_{m}/\tilde{r}\right)e^{{\tilde{\eta }}^{0}}+2{\tilde{D}}_{N}}{{\tilde{R}}_{p}{\tilde{R}}_{m}\ln\left({\tilde{R}}_{m}/{\tilde{R}}_{p}\right)e^{{\tilde{\eta }}^{0}}+2{\tilde{D}}_{N}}\right)K_{H}{\tilde{c}}^{0}\left(\tilde{x}\right)$$

Equation (15) allows us to calculate the flux ${\tilde{D}}_{N}\partial {\tilde{c}}_{N}^{0}/\partial \tilde{r}{|}_{\tilde{r}={\tilde{R}}_{p}}$:

(16) $${\left.{\tilde{D}}_{N}\frac{\partial {\tilde{c}}_{N}^{0}}{\partial \tilde{r}}\right|}_{\tilde{r}={\tilde{R}}_{p}}=-\frac{{\tilde{R}}_{m}{\tilde{D}}_{N}e^{{\tilde{\eta }}^{0}}K_{H}{\tilde{c}}^{0}\left(\tilde{x}\right)}{{\tilde{R}}_{p}{\tilde{R}}_{m}\ln\left({\tilde{R}}_{m}/{\tilde{R}}_{p}\right)e^{{\tilde{\eta }}^{0}}+2{\tilde{D}}_{N}}$$

With Equation (16), Equation (13) takes the form

(17) $${\tilde{D}}_{p}\frac{\partial^{2}{\tilde{c}}^{0}}{\partial {\tilde{x}}^{2}}=\rho {\tilde{c}}^{0},\;{\left.\frac{\partial {\tilde{c}}^{0}}{\partial \tilde{x}}\right|}_{\tilde{x}=0}=0,\;{\tilde{c}}^{0}\left(1\right)={\tilde{c}}_{1}^{0}$$

where

(18) $$\rho =\frac{2{\tilde{R}}_{m}{\tilde{D}}_{N}e^{{\tilde{\eta }}^{0}}K_{H}}{{\tilde{R}}_{p}\left({\tilde{R}}_{p}{\tilde{R}}_{m}\ln\left({\tilde{R}}_{m}/{\tilde{R}}_{p}\right)e^{{\tilde{\eta }}^{0}}+2{\tilde{D}}_{N}\right)}$$

The solution of Equation (17) reads

(19) $${\tilde{c}}^{0}\left(\tilde{x}\right)=\frac{{\tilde{c}}_{1}^{0}\cosh\left(\tilde{x}\sqrt{\rho /{\tilde{D}}_{p}}\right)}{\cosh\left(\sqrt{\rho /{\tilde{D}}_{p}}\right)}.$$

Using Equation (19) in Equation (15) and setting $\tilde{r}={\tilde{R}}_{m}$, we get ${\tilde{c}}_{N,m}^{0}$, which appears in Equation (9):

(20) $${\tilde{c}}_{N,m}^{0}=\frac{\rho {\tilde{c}}_{1}^{0}\cosh\left(\tilde{x}\sqrt{\rho /{\tilde{D}}_{p}}\right)}{e^{{\tilde{\eta }}^{0}}\cosh\left(\sqrt{\rho /{\tilde{D}}_{p}}\right)}.$$

For further calculations we need the polarization curve of the CCL. The steady-state proton current conservation equation in the CCL is

(21) $$\frac{\partial {\tilde{j}}^{0}}{\partial \tilde{x}}=-{\tilde{c}}_{N,m}^{0}\exp{\tilde{\eta }}^{0}$$

where ${\tilde{j}}^{0}$ is the local proton current density in the film. Integrating this equation over $\tilde{x}$ from 0 to 1 with Equation (20) and the boundary conditions ${\tilde{j}}^{0}\left(0\right)={\tilde{j}}_{0}$, ${\tilde{j}}^{0}\left(1\right)=0$, we find

(22) $${\tilde{j}}_{0}=\frac{{\tilde{c}}_{1}^{0}\rho \tanh\sqrt{\rho /{\tilde{D}}_{p}}}{\sqrt{\rho /{\tilde{D}}_{p}}}.$$

With ρ given by Equation (18), this equation provides the explicit dependence of ${\tilde{j}}_{0}$ vs. the ORR overpotential ${\tilde{\eta }}^{0}$, i.e., the static polarization curve of the system.

In two limiting cases, Equation (22) can be simplified. If the argument of the tanh-function is small, we may approximate tanhx≃x and Equation (22) simplifies to

(23) $${\tilde{j}}_{0}={\tilde{c}}_{1}^{0}\rho .$$

With ρ, Equation (18), we get expression of ${\tilde{\eta }}^{0}$ through ${\tilde{j}}_{0}$:

(24) $$e^{{\tilde{\eta }}^{0}}=\frac{2{\tilde{D}}_{N}{\tilde{R}}_{p}{\tilde{j}}_{0}}{{\tilde{R}}_{m}\left(2{\tilde{D}}_{N}K_{H}{\tilde{c}}_{1}^{0}-{\tilde{R}}_{p}^{2}\ln\left({\tilde{R}}_{m}/{\tilde{R}}_{p}\right){\tilde{j}}_{0}\right)},\;\mathrm{when}\;\sqrt{\rho /{\tilde{D}}_{p}}\ll 1$$

Equating the zero denominator of Equation (24) we get a limiting current density due to the oxygen transport in the Nafion film [20]

(25) $${\tilde{j}}_{N}^{\lim}=\frac{2{\tilde{D}}_{N}K_{H}{\tilde{c}}_{1}^{0}}{{\tilde{R}}_{p}^{2}\ln\left({\tilde{R}}_{m}/{\tilde{R}}_{p}\right)}.$$

Equations (24) and (25) do not contain ${\tilde{D}}_{p}$, meaning that this case corresponds to fast oxygen transport in the void pore.

In the limit of $\sqrt{\rho /{\tilde{D}}_{p}}\geq 2$, we may replace the tanh-function in Equation (22) by unity, which leads to

(26) $${\tilde{j}}_{0}=\frac{{\tilde{c}}_{1}^{0}\rho }{\sqrt{\rho /{\tilde{D}}_{p}}}.$$

With Equation (18) we find

(27) $$e^{{\tilde{\eta }}^{0}}=\frac{2{\tilde{D}}_{N}{\tilde{R}}_{p}{\tilde{j}}_{0}^{2}}{{\tilde{R}}_{m}\left(2{\tilde{D}}_{p}{\tilde{D}}_{N}K_{H}{\left({\tilde{c}}_{1}^{0}\right)}^{2}-{\tilde{R}}_{p}^{2}\ln\left({\tilde{R}}_{m}/{\tilde{R}}_{p}\right){\tilde{j}}_{0}^{2}\right)},\;\mathrm{when}\;\sqrt{\rho /{\tilde{D}}_{p}}\geq 2$$

Equation (27) determines the limiting current density

(28) $${\tilde{j}}_{N}^{\lim}=\sqrt{\frac{2{\tilde{D}}_{p}{\tilde{D}}_{N}K_{H}{\left({\tilde{c}}_{1}^{0}\right)}^{2}}{{\tilde{R}}_{p}^{2}\ln\left({\tilde{R}}_{m}/{\tilde{R}}_{p}\right)}}$$

Equation (27) correlates with the assumption of large oxygen transport loss in the CCL and below, this equation will be used in numerical calculations.

## 3. Results and Discussion

Static solutions of the previous section allow us to calculate the concentration impedance. The solution to Equation (9) is a rather cumbersome expression containing Bessel functions. This solution is only needed for calculation of the perturbed oxygen flux ${\tilde{N}}_{N,p}^{1}$ appearing in Equation (8). Rather tedious algebra leads to

(29) $${\tilde{N}}_{N,p}^{1}=\frac{{\tilde{D}}_{N}}{Q}\left(P_{c}K_{H}{\tilde{c}}^{1}\left(\tilde{x}\right)+P_{\eta }{\tilde{c}}_{N,m}^{0}\left(\tilde{x}\right)e^{{\tilde{\eta }}^{0}}{\tilde{\eta }}^{1}\right)$$

where the independent of $\tilde{x}$ coefficients $P_{c}$, $P_{\eta }$ and *Q* are given in Appendix A. Equation (29) with (20) allow us to solve the key Equation (8):

(30) $$\begin{array}{c} {\tilde{c}}^{1}\left(\tilde{x}\right)=\frac{2\rho P_{\eta }{\tilde{D}}_{N}{\tilde{c}}_{1}^{0}{\tilde{\eta }}^{1}}{{\tilde{R}}_{p}Q\cos\left(\phi \right)\left({\tilde{D}}_{p}\phi^{2}+\rho \right)\cosh\sqrt{\rho /{\tilde{D}}_{p}}}\\ \times \left(\cos\left(\phi \tilde{x}\right)\cosh\sqrt{\frac{\rho }{{\tilde{D}}_{p}}}-\cos\left(\phi \right)\cosh\left(\tilde{x}\sqrt{\frac{\rho }{{\tilde{D}}_{p}}}\right)\right)+\frac{{\tilde{c}}_{h}^{1}\cos\left(\phi \tilde{x}\right)}{\cos\phi } \end{array}$$

where

(31) $$\phi =\sqrt{\frac{2P_{c}{\tilde{D}}_{N}K_{H}}{{\tilde{R}}_{p}{\tilde{D}}_{p}Q}-\frac{i\tilde{\omega }}{{\tilde{D}}_{p}}}.$$

As discussed above, the model is valid in the limit of strong oxygen transport loss through the CCL depth. In this regime, the perturbation of oxygen concentration at the membrane surface is nearly zero: ${\tilde{c}}^{1}\left(0\right)≃0$. Setting in Equation (30) $\tilde{x}=0$, ${\tilde{c}}^{1}\left(0\right)=0$ and dividing the resulting equation by ${\tilde{c}}_{h}^{1}$, we get an algebraic equation for zeta-impedance

(32) $$0=\frac{2\rho P_{\eta }{\tilde{D}}_{N}{\tilde{c}}_{1}^{0}\tilde{\zeta }\left(\cosh\sqrt{\rho /{\tilde{D}}_{p}}-\cos\phi \right)}{{\tilde{R}}_{p}Q\left({\tilde{D}}_{p}\phi^{2}+\rho \right)\cosh\sqrt{\rho /{\tilde{D}}_{p}}}+1$$

Solving Equation (32) for $\tilde{\zeta }$, we finally find

(33) $$\tilde{\zeta }=\frac{{\tilde{R}}_{p}Q\left({\tilde{D}}_{p}\phi^{2}+\rho \right)\cosh\sqrt{\rho /{\tilde{D}}_{p}}}{2\rho P_{\eta }{\tilde{D}}_{N}{\tilde{c}}_{1}^{0}\left(\cos\phi -\cosh\sqrt{\rho /{\tilde{D}}_{p}}\right)}.$$

Equation (33) is the main result of this work.

**Figure 2:**
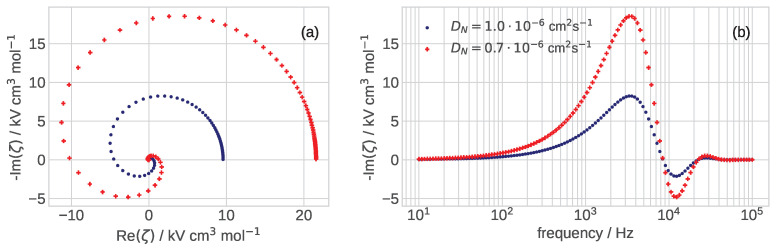
(**a**) The Nyquist spectra and (**b**) the frequency dependence of imaginary part of ζ-impedance, Equation (33), for the indicated values of oxygen diffusion coefficient in the Nafion film $D_{N}$, cm${}^{2}$ s${}^{-1}$. The other parameters for calculations are listed in Table 1.

The spectra of Equation (33) in the dimension form $\zeta =\tilde{\zeta }b/c_{ref}$ for the two oxygen diffusion coefficients $D_{N}$ in the Nafion film are shown in Figure 2. The base-case set of parameters used in the calculations is collected in Table 1. As can be seen, for these parameters, the zeta-spectrum is quite sensitive to the value of $D_{N}$ (Figure 2a), which makes zeta-spectroscopy a good candidate for measuring film transport properties. Note that the variation of $D_{N}$ does not change the characteristic frequency of the curve in Figure 2b.

**Figure 3:**
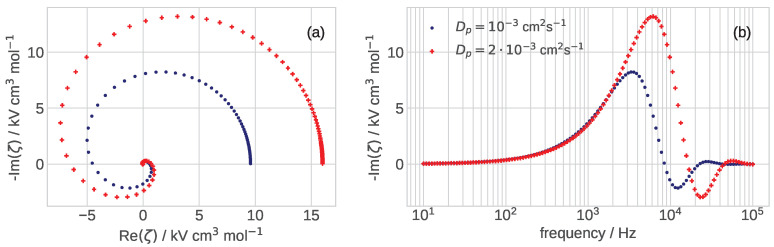
(**a**) The Nyquist spectra and (**b**) the frequency dependence of imaginary part of ζ-impedance, Equation (33), for the indicated values of oxygen diffusion coefficient in the pore $D_{p}$, cm${}^{2}$ s${}^{-1}$. The other parameters for calculations are listed in Table 1.

Variation of the zeta-spectrum with pore diffusivity $D_{p}$ is illustrated in Figure 3: the growth of $D_{p}$ increases the static “resistivity” of the spectrum and shifts the peak of −Imζ to higher frequency (Figure 3b). The dependence of −Imζ peak frequency on $D_{p}$ is close to linear: twice higher $D_{p}$ shifts the peak to twice larger frequency (Figure 3b). The characteristic frequency of peaks in Figure 3b is between 1 and 10 kHz, far above the characteristic frequency of faradaic processes in the cell, which typically does not exceed 100 Hz. It is interesting to note that variation of $D_{N}$ and $D_{p}$ lead to the opposite trends in the spectrum diameter: the curl increases with the decrease in $D_{N}$ and with the growth of $D_{p}$ (cf. Figure 2a and Figure 3a).

**Figure 4:**
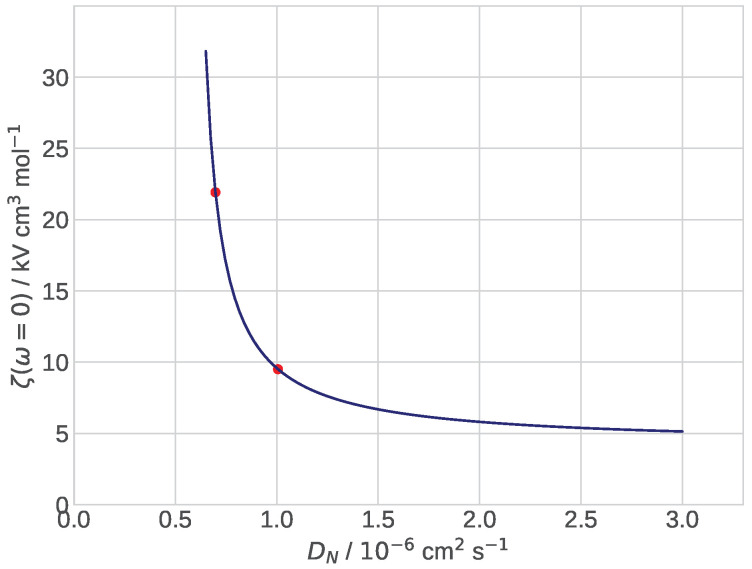
The dependence of static concentration impedance $\zeta^{0}$ on the oxygen diffusion coefficient in the Nafion film $D_{N}$. The spectra in Figure 2 are plotted at the points indicated by red dots.

Of particular interest is the static value ${\tilde{\zeta }}^{0}$, corresponding to the rightmost point of the spectra in Figure 2 and Figure 3. Unfortunately, Maple${}^{®}$ fails to calculate ${\tilde{\zeta }}^{0}=\lim_{\tilde{\omega }\to 0}\tilde{\zeta }$. Numerically calculated $\zeta^{0}$ as a function of the Nafion film diffusivity is shown in Figure 4. For $D_{N}$ below $1.0\times 10^{-6}$ cm${}^{2}$ s${}^{-1}$ the curve is very steep due to effect of limiting current: with the decrease in $D_{N}$, the right side of the dimension version of Equation (28) tends to the current of 1.5 A cm${}^{-2}$ fixed in the calculations and the zeta-impedance rapidly increases. From Figure 4 it follows that measurements of $D_{N}$ should be best done close to the limiting current $j_{N}^{\lim}$ due to oxygen transport in the film. In this range of currents, the zeta-impedance is most sensitive to Nafion film transport parameters.

The model above is developed assuming fast proton transport in the CCL. This means that the cell current density must be much less than the characteristic current for proton transport in the CCL:

(34) $$j_{0}\ll j_{*}=\frac{\sigma_{N}b}{l_{t}}$$

where $\sigma_{N}$ is the CCL proton conductivity. In a working PEMFC, $\sigma_{N}≃0.02$ S cm${}^{-1}$, hence with b=0.03 V and $l_{t}=3\times 10^{-4}$ cm we get $j_{*}=2$ A cm${}^{-2}$.

On the other hand, the condition ${\tilde{c}}_{1}^{1}≃0$ holds for the cell current density satisfying to

(35) $$j_{0}≳\frac{4FD_{p}c_{1}^{0}}{l_{t}}$$

while the largest sensitivity of the zeta-spectrum to the Nafion film oxygen diffusivity is achieved for the cell currents satisfying to

(36) $$j_{0}≃4Fc_{1}^{0}\sqrt{\frac{2D_{p}D_{N}K_{H}}{R_{p}^{2}\ln\left(R_{m}/R_{p}\right)}}$$

which is the dimension version of Equation (28). Equations (35) and (36) allow one to select optimal $c_{1}^{0}$ and $j_{0}$ for experiments.

## 4. Conclusions

In this study, a model for concentration (zeta-) impedance of the low-Pt cathode catalyst layer in a PEM fuel cell is developed. The model is based on the transient oxygen mass transport equations in the cylindrical pore surrounded by a thin Nafion film separating the pore volume from Pt/C surface. An analytical solution for zeta-impedance is obtained, which can be used for fast fitting experimental zeta-spectra. It is shown that the zeta-spectrum is very sensitive to the value of oxygen diffusion coefficient in the Nafion film, provided that the cell operates at a high rate of oxygen consumption in the CCL and close to the limiting current density due to oxygen transport in the film.

## Figures and Tables

**Table 1:** The base-case cell parameters used in calculations.

Tafel slope *b*, V	0.03
Exchange current density $i_{*}$, A cm${}^{-3}$	$10^{-3}$
Double layer capacitance $C_{dl}$, F cm${}^{-3}$	20
Oxygen diffusion coefficient in	
the Nafion film, $D_{N}$, cm${}^{2}$ s${}^{-1}$	≃$1\times 10^{-6}$
Dimensionless Henry’s constant for	
O${}_{2}$ solubility in water at 80 ${}^{\circ }$C, $K_{H}$	6.76 × $10^{-3}$
Catalyst layer thickness $l_{t}$, cm	$3\times 10^{-4}$ (3 μm)
Nafion film thickness $l_{N}$, cm	$10\times 10^{-7}$ (10 nm)
Pore radius $R_{p}$	$30\times 10^{-7}$ (30 nm)
Cell current density $j_{0}$, A cm${}^{-2}$	1.5
Pressure	Standard
Cell temperature *T*, K	273 + 80

## Data Availability

Data is contained within the article.

## Nomenclature

$\tilde{}$	Marks dimensionless variables
*b*	ORR Tafel slope, V
$C_{dl}$	Double layer volumetric capacitance, F cm${}^{-3}$
*c*	Oxygen molar concentration in the pore, mol cm${}^{-3}$
$c_{1}$	Static oxygen concentration at
	the CCL/GDL interface, mol cm${}^{-3}$
$c_{h}$	Oxygen concentration in the channel, mol cm${}^{-3}$
$c_{N}$	Oxygen concentration in the Nafion film, mol cm${}^{-3}$
$c_{N,m}$	= $c_{N}\left(R_{m}\right)$
$c_{ref}$	Reference (inlet) oxygen concentration, mol cm${}^{-3}$
$D_{b}$	Oxygen diffusion coefficient in the GDL, cm${}^{2}$ s${}^{-1}$
$D_{p}$	Oxygen diffusion coefficient in the pore, cm${}^{2}$ s${}^{-1}$
$D_{N}$	Oxygen diffusion coefficient in the Nafion film, cm${}^{2}$ s${}^{-1}$
*F*	Faraday constant, C mol${}^{-1}$
$i_{*}$	ORR volumetric exchange current density, A cm${}^{-3}$
i	Imaginary unit
*j*	Local proton current density along the pore, A cm${}^{-2}$
$j_{N}^{\lim}$	Limiting current density due to oxygen transport in Nafion film, A cm${}^{-2}$
$j_{*}$	Characteristic current density of proton transport, A cm${}^{-2}$, Equation (34)
$j_{0}$	Cell current density, A cm${}^{-2}$
$K_{H}$	Dimensionless Henry’s constant for oxygen solubility in Nafion, mol/mol
$l_{b}$	GDL thickness, cm
$l_{t}$	Pore length (CCL thickness), cm
$l_{N}$	Nafion film thickness, cm
$N_{N}$	Radial oxygen flux in the Nafion film, mol cm${}^{-2}$ s${}^{-1}$
*q*	Auxiliary parameter, Equation (A4)
$R_{m}$	Radius of a Pt/C tube, cm
$R_{p}$	Pore radius, cm
*r*	Radial coordinate, cm
*x*	Coordinate along the pore, cm
**Subscripts:**
0	Membrane/CCL interface
1	CCL/GDL interface
*h*	Channel
*m*	Pt/C (metal) surface
*N*	Nafion film
*p*	Pore/Nafion film interface
**Superscripts:**
0	Steady-state value
1	Small-amplitude perturbation
lim	Limiting

**Greek:**
γ	= $2/{\tilde{R}}_{p}$
η	ORR overpotential, positive by convention, V
ζ	Concentration impedance, V cm${}^{3}$ mol${}^{-1}$
ρ	Auxiliary dimensionless parameter, Equation (18)
$\sigma_{N}$	Nafion film proton conductivity, S cm${}^{-1}$
ϕ	Auxiliary dimensionless parameter, Equation (31)
$\tilde{\omega }$	= $\omega 4Fc_{ref}/i_{*}$, dimensionless frequency
ω	Angular frequency of the AC signal, s${}^{-1}$

## Appendix A. Coefficients in Equations (29)–(33)

(A1)
$$\begin{array}{c} P_{c}=\gamma q^{2}{\tilde{D}}_{N}(K_{1}\left(-q{\tilde{R}}_{m}\right)I_{1}\left(q{\tilde{R}}_{p}\right)-K_{1}\left(-q{\tilde{R}}_{p}\right)I_{1}\left(q{\tilde{R}}_{m}\right))\\ +qe^{{\tilde{\eta }}^{0}}(K_{0}\left(-q{\tilde{R}}_{m}\right)I_{1}\left(q{\tilde{R}}_{p}\right)-K_{1}\left(-q{\tilde{R}}_{p}\right)I_{0}\left(q{\tilde{R}}_{m}\right)) \end{array}$$

(A2)
$$P_{\eta }=q(K_{0}\left(-q{\tilde{R}}_{p}\right)I_{1}\left(q{\tilde{R}}_{p}\right)-K_{1}\left(-q{\tilde{R}}_{p}\right)I_{0}\left(q{\tilde{R}}_{p}\right))$$

(A3)
$$\begin{array}{c} Q=\gamma q{\tilde{D}}_{N}(K_{1}\left(-q{\tilde{R}}_{m}\right)I_{0}\left(q{\tilde{R}}_{p}\right)-K_{0}\left(-q{\tilde{R}}_{p}\right)I_{1}\left(q{\tilde{R}}_{m}\right))\\ +e^{{\tilde{\eta }}^{0}}(K_{0}\left(-q{\tilde{R}}_{m}\right)I_{0}\left(q{\tilde{R}}_{p}\right)-K_{0}\left(-q{\tilde{R}}_{p}\right)I_{0}\left(q{\tilde{R}}_{m}\right)) \end{array}$$

(A4)
$$q=\sqrt{\frac{i\tilde{\omega }}{{\tilde{D}}_{N}}},\;\gamma =\frac{2}{{\tilde{R}}_{p}}$$

Here, $I_{0}$, $I_{1}$ are the Bessel functions of the second kind, $K_{0}$, $K_{1}$ are the modified Bessel functions of the second kind.
